# Evaluating the gap in rapid diagnostic testing: insights from subnational Kenyan routine health data

**DOI:** 10.1136/bmjopen-2023-081241

**Published:** 2024-08-19

**Authors:** Bibian N. Robert, Angela K. Moturi, Felix Bahati, Peter M. Macharia, Emelda A. Okiro

**Affiliations:** 1 Population & Health Impact Surveillance Group, KEMRI-Wellcome Trust Research Programme, Nairobi, Kenya; 2 Health Services Research Unit, KEMRI-Wellcome Trust Research Programme, Nairobi, Kenya; 3 Department of Public Health, Institute of Tropical Medicine, Antwerpen, Belgium; 4 Centre for Tropical Medicine & Global Health, Nuffield Department of Medicine, University of Oxford, Oxford, UK

**Keywords:** EPIDEMIOLOGY, PUBLIC HEALTH, Health Services Accessibility

## Abstract

**Abstract:**

**Background:**

Understanding diagnostic capacities is essential to addressing healthcare provision and inequity, particularly in low-income and middle-income countries. This study used routine data to assess trends in rapid diagnostic test (RDT) reporting, supplies and unmet needs across national and 47 subnational (county) levels in Kenya.

**Methods:**

We extracted facility-level RDT data for 19 tests (2018–2020) from the Kenya District Health Information System, linked to 13 373 geocoded facilities. Data quality was assessed for reporting completeness (ratio of reports received against those expected), reporting patterns and outliers. Supply assessment covered 12 RDTs reported by at least 50% of the reporting facilities (n=5251), with missing values imputed considering reporting trends. Supply was computed by aggregating the number of tests reported per facility. Due to data limitations, demand was indirectly estimated using healthcare-seeking rates (HIV, malaria) and using population data for venereal disease research laboratory test (VDRL), with unmet need computed as the difference between supply and demand.

**Results:**

Reporting completeness was under 40% across all counties, with RDT-specific reporting ranging from 9.6% to 89.6%. Malaria RDTs showed the highest annual test volumes (6.3–8.0 million) while rheumatoid factor was the lowest (0.5–0.7 million). Demand for RDTs varied from 2.5 to 11.5 million tests, with unmet needs between 1.2 and 3.5 million. Notably, malaria testing and unmet needs were highest in Turkana County, as well as the western and coastal regions. HIV testing was concentrated in the western and central regions, with decreasing unmet needs from 2018 to 2020. VDRL testing showed high volumes and unmet needs in Nairobi and select counties, with minimal yearly variation.

**Conclusion:**

RDTs are crucial in enhancing diagnostic accessibility, yet their utilisation varies significantly by region. These findings underscore the need for targeted interventions to close testing gaps and improve data reporting completeness. Addressing these disparities is vital for equitably enhancing diagnostic services nationwide.

**STRENGTHS AND LIMITATIONS OF THIS STUDY**

- Subnational rapid diagnostic test (RDT) assessment in Kenya: The spatial analysis by county and RDT type reveals spatial heterogeneities in RDT availability for 12 communicable and non-communicable diseases.
- Supply landscape examination: Analyses of supply dynamics for RDTs identify distribution and accessibility challenges. We employed robust data quality checks and methods to account for missingness associated with District Health Information System (DHIS2) data.
- Identification of unmet needs: Applied indirect techniques leveraging routine and demographic data to highlight unmet RDT needs in vulnerable populations in settings where direct demand data are limited or unavailable.
- Supply underestimation: Possible underestimation of RDT supply due to data missingness from inconsistent health facility reporting.
- Unmet needs analysis: Analysis of unmet needs is restricted to only three tests due to limited availability of demand data.

## Introduction

 Access to diagnostic testing is vital for timely and appropriate healthcare provision.^1^ However, in many low-income and middle-income countries (LMICs), the availability of diagnostic services is inequitable and inadequate to meet current healthcare needs, with the largest deficit occurring at the primary care level.^2^,^4^ Yet the disproportionately high disease and mortality burden experienced in LMICs can be minimised or prevented by timely diagnosis.^5^ As the global agenda steers towards achieving universal health coverage (UHC), ensuring widespread and equitable diagnostic testing availability is a priority.^6^,^8^

Investment in diagnostics in Sub-Saharan Africa (SSA) has primarily been channelled towards essential programmes for HIV, tuberculosis (TB) and malaria,^9^ as evidenced by the greater availability of these tests in SSA.^10^ As these programmes are heavily donor-driven, asymmetries in diagnostic capacities for different conditions emerge, further complicated by the lack of dedicated funding for diagnostic testing within national healthcare budget allocations.^11^ A substantial shortfall in the availability of tests exacerbates unmet health needs within communities that face several barriers to accessing quality healthcare, including high poverty, poor physical access, inadequate human resources and limited health infrastructure.^12 13^ The deficit in diagnostic tests thus occasions provider reliance on syndromic diagnosis, which limits the accuracy of diagnoses, results in unnecessary treatments and affects the fidelity of disease surveillance data.^14 15^

The improvement and procurement of quality and affordable essential Health Products and Technologies (HPTs) are critical for the effective delivery of healthcare.^16^ Point-of-care tests, known as rapid diagnostic tests (RDTs), are a form of HPTs that can be used as entry points to reduce the diagnostic gaps across facilities in LMICs. The key advantages of RDTs are their low cost, ease of interpretation, low training and basic storage requirements, which provide a sustainable diagnostic solution.^17^ RDTs for HIV and malaria, which are widely available, have successfully increased evidence-based diagnoses within the same context.^18 19^ This has led to further investments in the development of diagnostic tools for various diseases and conditions. However, any investment case requires understanding the gaps in providing RDTs at the subnational level within a country.

To quantify gaps or unmet diagnostic needs, a comprehensive assessment of the current supply of RDTs and population groups that require access to a particular RDT is needed. However, a critical challenge persists due to the lack of data analyses to inform the targeted allocation of resources at the subnational level. To address this gap, this study aimed to assess and estimate the current supply, demand and unmet needs of various RDTs in Kenya using routine health data. The analysis was conducted at the national and subnational levels to inform health policies and resource allocation to improve diagnostic capacity and RDT development.

## Methods

### Settings

#### Country setting and healthcare system

This study was conducted in Kenya located in East Africa, with a population of approximately 47.6 million people according to the 2019 population census.^20^ Counties around Lake Victoria, central and coastal regions have the highest population densities whereas those in southern and northern parts are sparsely populated. Under the devolved healthcare system adopted in 2013,^21^ the national government is responsible for regulatory roles, including acquiring and maintaining strategic and special/expensive product stocks. In contrast, the 47 county governments (online supplemental figure S1) oversee health service delivery, ensuring the availability of essential/basic products.^22^

#### Diagnostic landscape

Investing in HPTs, such as diagnostic tests, is a primary focus of the Kenya Health Policy 2014–2030.^22^ Management guidelines have been developed to guarantee a constant supply of HPTs across all six levels of healthcare in the Kenyan community (level 1), dispensaries (level 2), health centres (level 3), primary hospitals (level 4), secondary hospitals (level 5) and tertiary hospitals (level 6).^23^ The public health laboratory system aligns with these levels, where levels 1–2 offer basic diagnostic services while level 5 has the highest diagnostic capacity within a county.^23^,^25^ Level 6 serves as a national reference referral where specimens are transferred from lower-level facilities.^26^ However, the majority of medical laboratory services are offered by the private sector (profit and non-profit).^27^

Health facilities must maintain a daily record-keeping system for commodities, whether paper based or electronic. These records are regularly compiled and reported to logistics managers and/or the health management information system (HMIS), such as District Health Information System Software (DHIS2), for decision-making. Bin/stock control cards and Daily Activity Registers are basic tools for managing commodities inventory.^23^ The Kenya Medical Supply Agency is mandated for procuring, warehousing and distributing essential medicine and medical supplies to counties and public health facilities.^28^ Faith-based facilities rely on the Mission for Essential Drugs and Supplies for their supply needs, whereas the private sector, though concentrated in urban centres, bridges the demand deficit.^16^ Procurement is guided by national legislation^29^ and regulations set by donor agencies in the case of priority programmes for HIV, malaria and TB.^16^

### Data

The number of diagnostic tests conducted at the facility level was extracted from DHIS2 between 2018 and 2020 using Ministry of Health (MoH) 706, a national laboratory summary reporting tool comprising 91 diagnostic tests. Daily diagnostic data from health facilities were aggregated monthly in the MoH 706 and entered in DHIS2. These monthly records were linked using the unique DHIS2 identifier to an updated geocoded health facility database,^4^ which includes a list of diagnostic health facilities.

#### Health facility reporting diagnostic tests in Kenya

There has yet to be a definitive list of health facilities that should report diagnostics tests in Kenya. Therefore, we indirectly assembled a pragmatic list of facilities expected to report RDTs in the country. We assumed that all health facilities should be reporting diagnostic tests, excluding medical stores, depots, treatment and rehabilitation centres.^4^ We leveraged a geocoded health facility database in Kenya to create this list and extracted 13 373 public and private health facilities. All facilities were included in the initial data quality assessment (RDT and facility completeness), and a subset of reporting facilities (those that submitted at least one test report), totalling 5231 (39.0%), was defined. This subset is a definitive list of active diagnostic testing centres and is considered reasonable for use in the assessment of RDTs reporting patterns and computation of supply, demand and unmet needs. Facilities with missing reports (blanks) across the entire study period were considered non-reporting, as we could not distinguish whether they represented true zeros or non-reporting or were due to a lack of diagnostic capacity. This challenge primarily arises from the design of the DHIS2 system whereby zero values entered in the system are converted to blank reports.^30^,^32^ A summary of the approach used in this study is shown in online supplemental figure S2.

#### Rapid diagnostic tests

We reviewed the assay formats outlined in the WHO Essential Diagnostic List (EDL) to identify clinical RDTs that support patient care. This EDL includes recommended clinical diagnostic tests that should be available at the point of care across all health tires.^33^ The EDL was complemented with local clinicians’ knowledge, and 19 RDTs (online supplemental table S1) were compiled from the 91 diagnostic tests reported in the MoH 706 form. These tests were further reclassified as either common or uncommon, by level expected to be reported, and by WHO EDL categories, as detailed elsewhere.^4 30^ Monthly data from 2018 to 2020 for each of the 19 RDTs were downloaded at the facility level using the MoH authorised login^34^ on 25 November 2021. We chose this timeline to account for reporting biases in certain months caused by disruptive events such as health worker strikes,^35^ facility stockouts^36^ and the COVID-19 pandemic.^37^

#### Demand-related datasets

Datasets on population distribution of pregnant women, healthcare-seeking and disease testing rates that were required to estimate the demand for a subset of the RDTs include:

- HIV testing rates for adults: The proportion of adults and adolescents (15–64 years) who had been tested for HIV based on a nationally representative population-based survey of Kenya AIDS Indicator Survey.^38^
- Fever-treatment-seeking rate: The percentage of children under age 5 with fever in the 2 weeks preceding the survey for whom advice or treatment was sought based on the 2020 Malaria Indicator Survey (MIS).^39^
- Antenatal care (ANC) attendance rate by skilled provider: The percentage of women who had a live birth in the 2 years preceding the survey who received ANC during pregnancy for the most recent live birth from a skilled provider (doctor, nurse/midwife or health personnel), MIS 2020.^40^
- Total number of pregnant women at the county level from WorldPop.^41^

### Analysis

#### Data quality assessment

Routine data are subject to data quality and completeness considerations^41 42^ and we assessed the quality of the RDT data before estimating supply and demand. This process was guided by the WHO Data Quality Review Framework.^42^ Specifically, we examined the following:

- Reporting completeness (n=13 373)
  1. Facility reporting completeness: The ratio of reports received against those expected for all facilities (n=13 373), irrespective of RDT, disaggregated by facility levels. The expected reports were computed as the total number of facilities expected to report multiplied by the study period (36 months).
  2. RDT reporting completeness: We repeated the step in (1) but calculated the rate individually for each of the 19 RDTs.
- The proportion of reporting facilities (n=5251) reporting each RDT: The number of facilities reporting a particular RDT divided by all reporting facilities annually and over the 3 years. All RDTs used the same denominator, except for community health extension worker (CHEW) Malaria RDTs, which were limited to 10 counties in western Kenya (Bungoma, Busia, Homa Bay, Kakamega, Kisumu, Kisii, Migori, Nyamira, Siaya and Vihiga).
- Reporting patterns: Based on step (b) results, we assessed health facility reporting patterns for 12 RDTs reported by at least 50% of the health facilities across all levels. This was because of the high rate of non-reporting by health facilities; thus, a pragmatic 50% cut-off was applied. For each RDT, we categorised facilities into two groups: proportion reporting nine or more months of data and those reporting 1–8 months of data per year.
- Outliers: defined as median absolute deviations (MAD)—the median of the absolute value of each data point’s deviation—from the facility median for each RDT over 36 months. Outliers were first identified using the MAD approach^43^ investigated and treated as missing prior to computing supply. An example of outliers identified is shown in online supplemental figure S3.

### Estimating the supply of RDTS

Supply here is defined as the number of rapid tests performed by health facilities in Kenya, calculated as the number of tests reported per facility (aggregated across subnational units and nationally over time). We computed estimates of supply for 12 RDTs that met the 50% threshold. These tests included HIV, VDRL (venereal disease research laboratory test), urine chemistry, HCG (human chorionic gonadotropin), blood sugar, malaria RDT, *Helicobacter pylori*, blood group, HB (haemoglobin), rheumatoid factor, brucella and CHEW malaria RDT.

#### Addressing missing data

Data were categorised into two subsets of reporting facilities: those reporting at least 9 months of diagnostic data per year (type 1) and those reporting 1–8 months of diagnostic data (type 2) per year. Type 1 and type 2 facilities were systematically different in terms of the volumes reported. We, therefore, used different approaches to account for non-reporting to avoid bias on the adjustment of type 2 facilities. For type 1, we used the average value before (pre) and after (post) the missing to fill in for the non-reported month. For type 2, we took each facility’s average of all reported months (1–8 months) and used the resulting average monthly value for non-reported months. The summation of adjusted types 1 and 2 was the supply among the reporting facilities and was summarised nationally and subnationally across 47 counties over the 3 years.

### Estimating demand and unmet need

Data on populations that required diagnostic testing were not directly available. Thus, we used three tests, HIV, VDRL and total malaria RDT, as exemplars to estimate demand and unmet needs. This is because these tests had indirect data that presented minimal ambiguity in demand estimation.

Malaria RDTs: We used the health-seeking rates of symptomatic cases in a community visiting a health facility for treatment after a fever. We assumed this proportion represented the number of tests conducted at the facility. Therefore, those who did not reach a health facility but would have required a test represented an unmet indirect demand in the community. Thus, we derived the demand for malaria RDTs for each county, by dividing the total malaria RDTs supply by the proportion of under-5 with fever that sought care (equation 1). These rates were available by malaria risk zones, and a uniform value was used across all counties within each risk zones (online supplemental table S2). We assumed that fever care-seeking rates of children under-5 years of age were similar to adult fever care-seeking rates due to a lack of data.^44^ Furthermore, for health facilities in Western Kenya, we included malaria RDTs conducted by community health volunteers (CHEW Malaria RDTs) to account for the community case management of malaria which supplements facility-based healthcare in the region.

Equation 1. Estimating demand using health care-seeking rate.

(1) $$Demand=\sum_{1}^{n}X_{ij}/Y_{ij}\;\%$$

*where n is the total number of health facilities, X is total supply adjusted foroutliers* ∧ *non-reporting,* ∧* Y % is the health care seeking rate for county _i_* ∧* RDT j*.

HIV RDTs: We used the HIV testing rate for individuals who had ever been tested for HIV, most of whom had their HIV tests conducted at a health facility. We assumed that the proportion who underwent testing represented the number of HIV tests reported in the DHIS2 and reflected the demand. Thus, to calculate the demand for each county, we first matched the provincial HIV testing rate for people aged 15-64 with the corresponding counties within the same province (online supplemental table S2). We then divided the total RDTs supply by the county HIV testing rates (equation 1).

VDRL tests:VDRL tests are essential for ANC. To estimate demand, we gathered pregnant women’s data by county. We factored in the non-pregnant population needing the test, using DHIS2 data showing that 80% of the tests were ANC-related. We created scaling factors for each county, adjusting for non-ANC demand (equation 2). The factor was applied to pregnant women with ANC visits obtained by multiplying ANC attendance rate by skilled provider by the total pregnant population. For instance, if ANC comprised 70% of the tests in a county, the non-ANC demand would be 30% of ANC attendees. This was added to the total pregnant population to compute the final VDRL demand.

Equation 2: estimating VDRL demand

(2) $$VDRL\;Demand=P_{i}+\frac{\left(A_{i}\%\;*P_{i})\;*(1-R\right)}{3}$$

*where i is the county, P representsthe pregnant population, A%isthe ANC attendance rate (ANC by skilled provider/recent live births in the last2yearspreceding MIS 2020 survey×100), Risthe average ratio of ANC VDRL test tototal VDRL, (1−R) is the scaling factor divided by 3 to represent an average of3years.*

#### Unmet need for RDT

Unmet needs were then derived as the difference between supply and demand and assessed at the national and subnational levels.

Data analysis was conducted in R software environment (V.4.3) and StataCorp. 2021 (Stata Statistical Software: Release V.17., StataCorp). The results were mapped in ArcGIS Pro V.3.0.3 (ESRI, Redlands, California, USA).

### Patient and public involvement

None.

## Results

### Spatial variation of facilities and RDT reporting completeness across all facilities (n=13 373)

**Figure 1:**
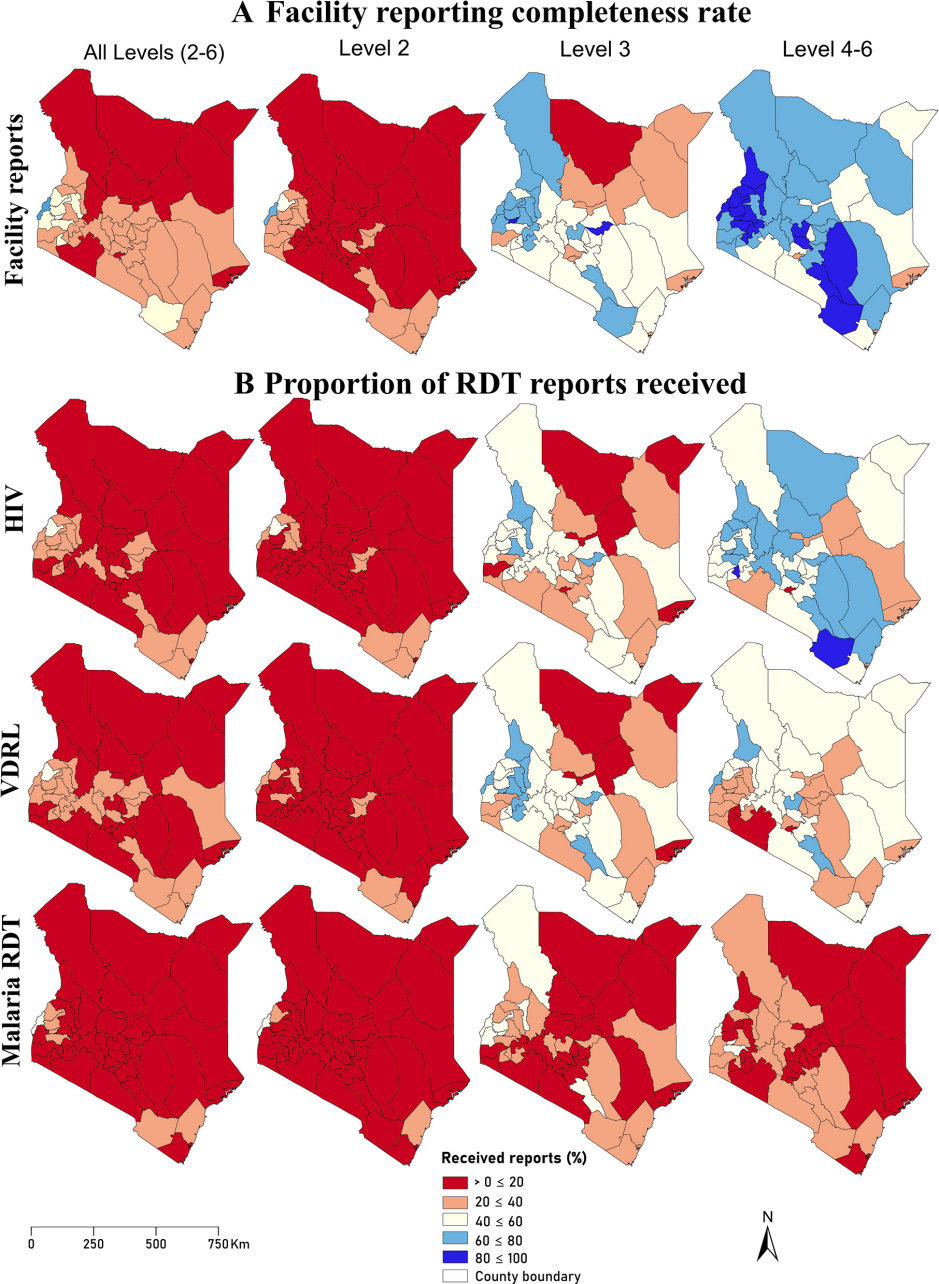
Geographical variation in the proportion of reports received from those expected to be submitted across the study period (2018–2020) by level. (**A**) The proportion of reports received^1^ from health facilities (irrespective of RDT) by county out of expected^2^ reports facility reports. (**B**) The proportion of RDT reports received for a subset of the RDTs considered: HIV, VDRL and malaria RDTs out of expected RDT reports. ^1^Received reports refer to MoH 706 reports containing rapid diagnostic data. ^2^Expected reports refer to the number of monthly reports required to be submitted within a specific timeframe in the DHIS2. Since this study spans 3 years, it means each facility is expected to have submitted 36 reports. DHIS2, District Health Information System; RDT, rapid diagnostic test; VDRL, venereal disease research laboratory; MoH, Ministry of Health.

Nationally, the average facility reporting completeness was 28.7% (95% CI 25.1% to 32.3%). Across the 47 counties, the facility completeness varied from 9.5% in Marsabit to 70.4% in Busia county, with 38 (80.9%) counties recording rates below 40% (figure 1A). Notably, counties with completeness below 20% were mainly concentrated in the northern region while those above 40% were mainly located in the western region. Further disaggregation of facility completeness by health facility level revealed that for level 2 facilities, completeness varied from 3.7% in Marsabit to 66.6% in Busia counties. Level 3 facilities exhibited a range from 14.7% in Marsabit to 82.3% in Tharaka-Nithi and in level 4–6 facilities, completeness varied from 21.5% in Busia to 95.6% in West Pokot.

RDT-specific reporting completeness is shown in figure 1B for a subset of tests (HIV, VDRL and Malaria RDTs). The remaining tests are shown in online supplemental figure S4. The completeness varied across counties, by levels and by specific RDTs. When all health facilities were considered, reporting completeness was below 40% across the three RDTs in all counties, except in Bungoma where completeness was 47.7% and 44.5% for HIV and VDRL, respectively, and in Busia where malaria RDT was 46.7%. Across level 2 facilities, over 75% of the counties reported below 20% across the three RDTs. The highest completeness for HIV and VDRL was in Bungoma at 42.1% and 37.8%, respectively, and 43.9% in Busia County for Malaria RDT. Across level 3 facilities, completeness ranged from 12.4% in Marsabit to 76.1% in Tharaka-Nithi. For VDRL, rates varied between 11.5% in Marsabit and 74.5% in Tharaka-Nithi while for Malaria RDT, rates ranged from 4.14% in Kirinyaga to 59.1% in Busia. For level 4–6 facilities reporting completeness ranged from 12.5% in Nairobi to 86.4% in Nyamira, 15.5% in Nairobi to 91.7% in West Pokot for VDRL and 2.2% in West Pokot to 49.07 in Vihiga for malaria RDT. The reporting completeness pattern for the other 16 RDTs (online supplemental figure S4) was also below 40% for the majority of RDTs, with an increase in completeness observed across higher facility levels. However, in some RDTs such as oral glucose tolerance test (OGTT), completeness remained below 20% even at higher facility levels.

### Summary statistics of 12 RDTs for reporting facilities (n=5251)

Table 1 lists the 12 RDTs selected for further analysis, including community (CHEW malaria) and facility malaria RDTs that were consistently reported by at least 50% of the reporting facilities (submitted at least one test in 3 years). Over the 3 years, the overall proportion of facilities reporting each RDT ranged between 50.9% for CHEW Malaria RDTs to 89.6% for HIV tests. The reporting rates for the seven RDTs that did not meet the 50% threshold ranged from 9.6% OGTT to 44.4% for antistreptolysin O titre test and are shown in online supplemental table S3.

**Table 1:** Summary of the 12 RDTs reported on the MoH 706 tool of Kenya’s health management information system (DHIS2).

RDTs	Reporting facilities, No. (%)*			
	2018–2020 (n=5251)|||||||||||||||||||||||||||||||||||||||||||||||||||| 100.0%	2018 (n=4381)	2019 (n=4656)	2020 (n=4863)
HIV	||||||||||||||||||||||||||||||||||||||||||||||| 89.6%	3779 (86.3%)	4045 (86.9%)	4291 (88.2%)
Venereal disease research laboratory	|||||||||||||||||||||||||||||||||||||||||||||| 88.7%	3779 (86.3%)	4045 (86.9%)	4300 (88.4%)
Urine chemistry	|||||||||||||||||||||||||||||||||||||||||||||| 87.8%	3790 (86.5%)	4027 (86.5%)	4265 (87.7%)
Human chorionic gonadotropin	||||||||||||||||||||||||||||||||||||||||||||| 85.7%	3582 (81.8%)	3890 (83.5%)	4158 (85.5%)
Blood sugar	|||||||||||||||||||||||||||||||||||||||||||| 85.4%	3559 (81.2%)	3827 (82.2%)	4120 (84.7%)
Malaria RDT	||||||||||||||||||||||||||||||||||||||||||| 82.8%	3268 (74.6%)	3432 (73.7%)	3396 (69.8%)
*Helicobacter pylori*	|||||||||||||||||||||||||||||||||||||||||| 81.7%	3355 (76.6%)	3640 (78.2%)	3851 (79.2%)
Blood group	|||||||||||||||||||||||||||||||||||||||||| 80.4%	3346 (76.4%)	3549 (76.2%)	3828 (78.7%)
HB estimation	||||||||||||||||||||||||||||||||||||||||| 78.7%	3184 (72.7%)	3468 (74.5%)	3673 (75.5%)
Rheumatoid factor	||||||||||||||||||||||||||||||||||||||||| 78.6%	3259 (74.4%)	3523 (75.7%)	3603 (74.1%)
Brucella	||||||||||||||||||||||||||||||||||||| 70.5%	2816 (64.3%)	2973 (63.9%)	2990 (61.5%)
CHEW malaria RDTs†	|||||||||||||||||||||||||| 50.9%	712 (54.8%)	707 (52.0%)	694 (49.0%)

(n) represents the total number of reporting facilities.

* The proportion of reporting facilities per RDT was computed per year and across 36 months months as the number of facilities reporting RDT out of those reporting test volumes >0 (reporting facilities).. *** CHEW Malaria RDTs the denominator (n) is the number of reporting facilities across 10 western Kenya counties.

† CHEW malaria RDTs the denominator (n) is the number of reporting facilities across 10 western Kenya counties.

CHEWCommunity Health Extension WorkerDHIS2District Health Information SystemHBhaemoglobinMoHMinistry of HealthRDTrapid diagnostic test

### RDT reporting patterns among facilities reporting at least once (n=5251)

**Figure 2:**
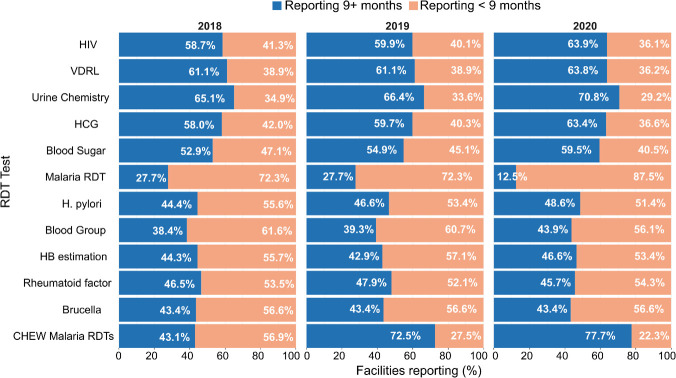
Proportion of facilities reporting ≥9+ months and <9 months of RDT data per year among those reporting an RDT (shown in table 1). The denominator for each RDT varies annually, as shown in table 1. HB, haemoglobin; HCG, human chorionic gonadotropin; RDT, rapid diagnostic test; CHEW, community health extension worker

Of the 12 RDTs analysed, HIV, VDRL, urine chemistry, HCG and blood sugar had more than 50% of the facilities reporting data for nine or more months each year. In contrast, over 70% of facilities reported less than 9 months of data annually for facility Malaria RDTs. The proportion of facilities reporting 9+ months of data for CHEW malaria RDTs increased from 43.1% in 2018 to 77.7% in 2020 (figure 2). Conversely, for facility malaria RDT tests, only 12.5% of the facilities were reporting 9+ months of tests by 2020, a decrease from 27.7% in 2018. For the other five tests, the proportion of facilities with at least 9 months of data ranged between 38.4% and48.6% with minimal variation observed across the years (figure 2).

### RDT supply

**Figure 3:**
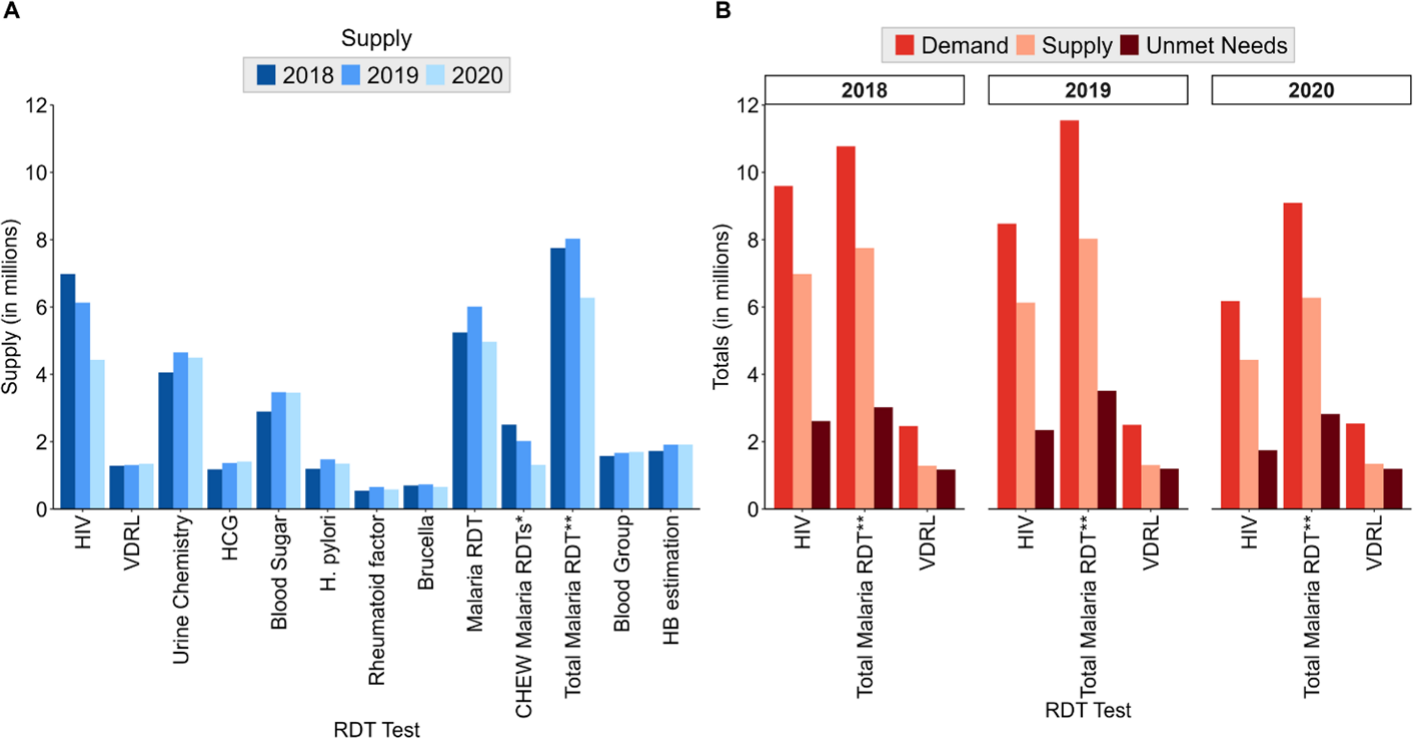
(**A**) Annual supply of 12 RDTs at the national level for facilities that submitted at least one report. (**B**) Annual supply, demand and unmet needs for three RDTs (HIV, total Malaria RDTs and VDRL) only due to data limitations. *Supply restricted to 10 CHEW counties in western Kenya. **Total Malaria RDT computed as the sum of CHEW Malaria RDTs and facility Malaria RDTs. RDTs, rapid diagnostic tests; VDRL, venereal disease research laboratory; CHEW, community health extension worker.

In this study, we provide a detailed analysis of the annual supply estimates for 12 RDTs across 5251 reporting facilities from 2018 to 2020 (figure 3A). Among these, malaria RDTs, including CHEW malaria RDTs and facility malaria RDTs, were the most frequently conducted, with annual totals reaching approximately 8 million tests in 2018 and 2019, and a decrease to around 6 million tests in 2020 (ranging from 6 274 316 to 8 028 315 tests). In contrast, the rheumatoid factor test was the least used, with annual figures between 546 956 and 653 261 tests.

While HIV and CHEW malaria RDTs experienced a consistent decline over the observed period, the demand for other RDTs generally increased, as shown in figure 3A. Despite this trend, post-2019 data indicate a reduction in testing volumes for several RDTs, most notably in Malaria RDTs, with the largest decrease observed in this category. This decline contrasted with the increases in test volumes for specific RDTs such as VDRL, blood grouping and HCG, which saw rises in tests performed in 2020.

### Demand and unmet needs estimates

The annual national demand and unmet needs for HIV, malaria and VDRL RDTs are shown in figure 3B. Among these RDTs, total malaria RDTs had the highest estimated yearly demand, ranging from 9 094 619 to 11 541 712 tests and the greatest testing gap ranging from 2 820 305 to 3 513 397 tests. The estimated demand for malaria RDTs outstripped that of HIV tests by 12.2%–47.1%, with the demand for HIV tests spanning from 6 180 640 to 9 592 672. Additionally, malaria RDTs exhibited 15.6%–61.4% greater unmet needs compared with HIV. In comparison, the annual demand and unmet needs for VDRL tests constituted about a third (2.5 million) and less than half (approximately 1.2 million tests), respectively, of the total for Malaria RDTs.

From 2018 to 2020, the estimated demand and unmet needs for HIV tests saw a gradual decline of 35.6% and 33.1%, respectively. In contrast, estimated malaria RDT demand initially rose by 7.2% in 2019, then fell by 21.2% in 2020. Unmet needs for malaria RDTs increased by 16.3% in 2019 before dropping by 19.7% in 2020. Meanwhile, VDRL test demand grew modestly at about 1.5% annually, from 2 463 908 in 2018 to 2 536 865 in 2020, with unmet needs peaking at 1.8% in 2019 before a slight decrease of 0.2% in 2020 (figure 3B).

### Spatial variation of supply, demand and unmet need

**Figure 4:**
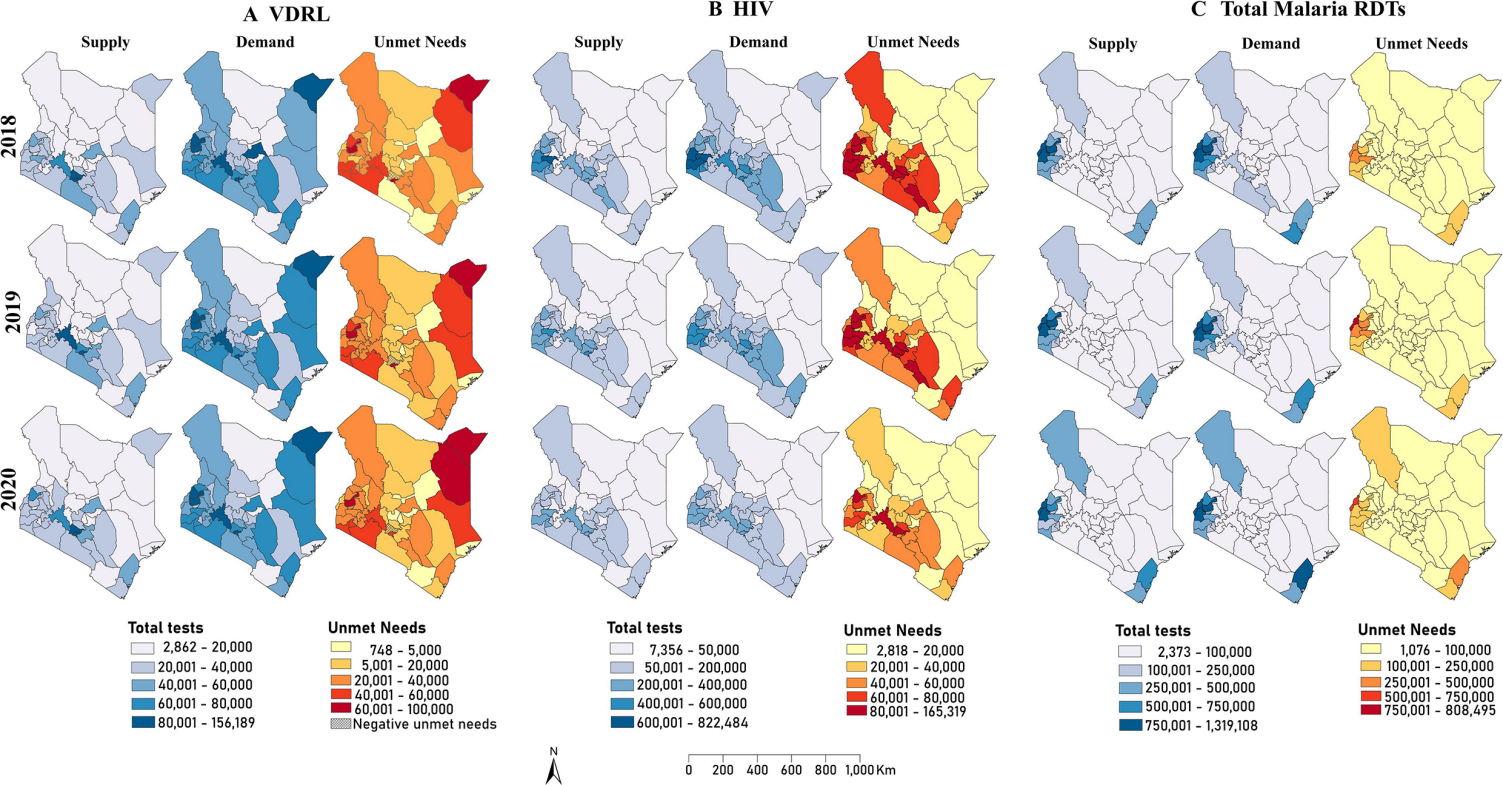
Geographical variation in supply, demand and unmet needs for (A) VDRL, (B) HIV, and (C) Total malaria RDTs per year among all reporting facilities. RDTs, rapid diagnostic tests; VDRL, venereal disease research laboratory; HIV, human immunodeficiency virus.

The supply, demand and unmet needs for VDRL, HIV and total malaria RDTs varied across counties, with minimal variations observed between years (figure 4). The supply for the other eight RDTs is shown in online supplemental figure S5A,B.

VDRL test volumes varied across counties from 2862 (Lamu) to 112 386 (Nairobi) (figure 4A). Kiambu, Nairobi and Nakuru counties conducted over 60 000 tests annually. Northern and northeastern counties except for Mandera and Garissa conducted tests below 20 000. Other counties reporting below 20 000 tests were distributed along the coast, eastern, central regions. Demand for VDRL tests ranged from 5073 (Lamu) to 156 189 (Nairobi). Bungoma, Kakamega, Nairobi, Nakuru and Mandera consistently had VDRL demand, exceeding 95 000 tests annually. Conversely, the annual demand in Samburu, Marsabit, Tharaka-Nithi, Taita Taveta, Isiolo and Lamu remained below 20 000 tests. The unmet needs for VDRL tests across counties mostly fell between 20 000 and 40 000 tests, accounting for 38% of the counties in 2018 and 2020 and 42% in 2019. Counties such as Mombasa (2018) and Kiambu (2018–2020) counties had negative unmet needs. Others like Kajiado and Wajir experienced an increase in unmet needs over the years.

HIV test volumes varied significantly across Kenyan counties, ranging from 7356 in Lamu to 657 165 in Kisumu. Notably, western Kenyan counties—Kisumu, Siaya and Bungoma—saw more than 400 000 HIV tests in 2018 and 2019 (figure 4B). Other regions, including Nakuru, Nairobi, Kiambu, Murang’a and Makueni, also reported substantial testing volumes, between 200 000 and 400 000 tests. In contrast, most counties in northern and northeastern Kenya recorded fewer than 50 000 tests, with Turkana being a notable exception. By 2020, there was a noticeable decrease in HIV testing volumes, with only six counties reporting over 200 000 tests. The demand for HIV tests in 2018 and 2019 was particularly high in western, central and eastern counties, with Turkana and Mandera leading in the northern region. The gap in HIV testing, which decreased over time, ranged from 2818 in Lamu to 165 319 in Kisumu, with counties such as Busia, Nakuru and Murang’a consistently exhibiting significant gaps of over 80 000 tests.

The demand for Malaria RDTs varied widely, from as few as 3449 tests in Wajir to a peak of 2 127 603 in Busia, where the most substantial testing gap of 808 495 was also recorded. Overall, 75% of counties (n=35) conducted fewer than 100 000 Malaria RDT tests. Notably, counties in western Kenya, along with Turkana in the northern region and Kilifi and Kwale on the coast, recorded the highest testing volumes. Busia, Siaya and Kakamega stood out, each conducting over 750 000 tests (figure 4C) indicating significant demand and unmet needs for malaria RDTs in these areas. In contrast, the majority of counties (75%) reported much lower testing figures (<100 000). Both the highest and lowest demands, along with the corresponding testing gaps, were noted in 2019. By 2020, while Busia County continued to lead in demand and testing gap, these figures had decreased to 1 437 121 and 541 661, respectively.

## Discussion

Assessments of diagnostic test availability and unmet needs across a spectrum of diseases within countries over time are lacking in low-resource settings. This study examined the supply, demand and testing gaps in RDTs using data from Kenya’s health information system between 2018 and 2020. The findings show the number of RDTs performed, the demand and extent of unmet needs at the national level and heterogeneity across subnational units used for decision-making. Importantly, these findings suggest that no specific region is systematically disadvantaged in terms of RDT availability. However, the observed heterogeneity across subnational units highlights the importance of tailored approaches that prioritise subnational units with the highest unmet needs, where access to RDTs is most needed thereby informing more effective resource allocation.

Less than half of all the registered facilities provided comprehensive diagnostic reporting, resulting in significant gaps in the data (figure 1A). Furthermore, the rate at which facilities submitted reports varied widely from 9.6% to 89.6% over the 3 years (table 1). This discrepancy in reporting can, in part, be attributed to variations in disease prevalence across different counties, which affects testing frequency and, consequently, reporting. However, it is crucial to highlight that HIV, malaria and VDRL tests, which have been the focus of long-term disease control efforts, exhibited higher reporting and data completeness levels than the other tests. Compared with HIV and malaria, VDRL stands out given that syphilis prevalence in the general population in Kenya is quite low at approximately 1.8%.^45^ However, better reporting is driven by the fact that it is a vital component of ANC and thus routinely conducted among pregnant women. The intensified attention given to these diagnostic tests (HIV, malaria and VDRL) appears to have improved data collection practices over time to support informed programmatic decisions. However, the unequal emphasis on diagnostic reporting among facilities poses a significant limitation to the robustness of surveillance systems, hindering the ability to track disease trends and assess the effectiveness of interventions over time.

Malaria testing accounted for the highest volume of tests across all 3 years, primarily driven by counties in the western region of Kenya, where malaria prevalence is highest.^46 47^ The greater volumes of malaria testing could be linked to the hybrid approach employed in malaria control, in which RDT testing is performed at the facility and within the community. However, it is noteworthy that despite this extensive testing, significant gaps exist in addressing the malaria burden in certain counties, such as Busia and Kilifi, where testing falls short over successive years compared with other regions. This reveals that significant gaps persist in effectively diagnosing populations at risk of disease despite substantial investments and long-standing interventions. These findings suggest the need for a more nuanced strategic approach to malaria control in these regions.

The utility of community-level testing in improving case detection and reducing the presumptive use of treatments has been elaborated in the literature.^48 49^ However, significant drawbacks in case management were experienced in areas with high malaria incidence following a court ruling in 2019 that prohibited the use of RDTs by community health workers.^50^ This ruling resulted in a noticeable decline in malaria testing within the community and a simultaneous increase in facility-based testing in 2019 (figure 3A). Although community-level initiatives eventually resumed, the gap in testing due to the ruling led to missed opportunities for malaria control within communities at the greatest risk. This interruption underscores the importance of addressing barriers to accessing diagnostic testing to effectively control different diseases within the population.

Over 3 years, the data revealed a consistent upward trend in the annual testing volumes across most RDTs. Notably, the most significant surge in testing occurred between 2018 and 2019 while 2020 saw these numbers stabilising for most tests. This pattern mirrors the expected rise in demand owing to population growth and improved reporting practices across various healthcare facilities, as illustrated in figure 3. However, two exceptions to this trend stand out: HIV and community-based malaria testing (CHEW), which experienced a continuous decline throughout the 3 years (figure 3A). Community-based malaria testing faced setbacks due to testing limitations mentioned earlier, compounded by the impact of the COVID-19 pandemic in 2020. This led to reduced community testing because of the isolation measures implemented to curb the spread of the virus.^37^ The unique dip in HIV testing rates may be attributed to interventions that have promoted greater awareness and accessibility of self-testing options, factors not accounted for in this analysis.^51 52^ Despite the growing range of testing choices, the persistent gap in HIV status awareness remains concerning, with as many as 20% of the population never having undergone testing in 2019.^52^ This emphasises the urgent need for intensified efforts in facility-based, community testing and self-testing interventions to sustain the progress made in disease control thus far.

Unmet needs across the years were heterogeneous across counties, with decreases and increases in testing gaps. This variation in trends has significant implications for addressing healthcare inequalities across diverse populations. The most significant decline in the unmet need for VDRL is in central Kenya, which has historically had better health services and infrastructure.^53^ Conversely, increases in unmet needs are experienced in the northern and partly coastal regions of Kenya, characterised by marginalisation and recurring conflicts.^54^ Ensuring access to diagnostics in these marginalised regions will contribute the most to the UHC agenda. The estimates presented in this study serve as a crucial baseline for the ongoing monitoring of unmet healthcare needs, thereby facilitating evidence-based policy interventions.

Assessments of the current diagnostic landscape within SSA are few and primarily focused on HIV and malaria diagnostic capacities in public health facilities.^55^ These evaluations used data from nationally representative service provision assessments and in-person surveys.^50 56 57^ These are limited to the scope of the considered diagnostic test, the generalisability of findings across various geographical scales and the need for resources for implementation. As countries seek to increase their diagnostic capacity, routine data will provide a sustainable data solution at the national and subnational levels. However, routine data currently requires improved reporting. For example, in Kenya, this barrier was notable in primary care health facilities with the poorest reporting rates despite being the majority (figure 1). Even though we accounted for non-reporting in the data, this is not a replacement for quality data. There remains uncertainty about why health facilities fail to submit reports even when they can perform tests.^4^ However, this may, in part, be due to stockouts and limited health resource capacity. Further, the ambiguity introduced by the lack of ‘zero’ reports in DHIS2^30^,^32^ may lead to an overestimation of the supply of RDTs and an underestimation in reporting by facilities. Further research is needed to better understand the barriers to reporting to reliably address unmet needs.

The continued use and implementation of RDTs for multiple conditions requires significant effort to ensure that tests are physically accessible and affordable. To ensure the uptake and utilisation of rapid tests, it is necessary to overcome barriers to healthcare-seeking and testing habits within populations to effectively deliver prompt diagnostic services, thereby ensuring early and accurate detection of diseases. Additionally, it is critical to improve the granularity and completeness of routine and survey data, specifically for diagnostics across various population groups. Coupled with fine scale and up-to-date prevalence data to identify disease hotspots, these data would help to establish target populations that require a test with greater precision.

## Limitations

This study has some limitations that should be considered when interpreting the findings. We approximated demand based on testing and healthcare-seeking rates obtained from routine and survey data owing to the absence of specific population data to approximate the demand. Although this is a practical approach, it is compounded by several ambiguities. The estimates of healthcare-seeking rates are based on surveys comprising small samples that may fail to reach those underserved or unreached by the health system. Furthermore, these sources typically focus on select tests and populations of interest such as children, resulting in estimates that may not reflect the health-seeking behaviours of the entire population that needs testing. Additionally, the care-seeking rates were applied to routine data, which has several quality and completeness limitations and thus may result in both overestimations and underestimations of demand and unmet needs The structure of DHIS2, which converts zero entries to missing values,^30^,^32^ can introduce bias into supply estimates, both underestimating and overestimating them. This issue stems from poor reporting practices and the system’s inability to distinguish between non-reporting facilities and those that genuinely conduct no tests in a given period, thereby implying a true ‘zero.’ Such ambiguity affects the accuracy of supply estimates, as facilities with the capacity to test might either neglect to report data for periods when tests are not conducted or fail to submit reports altogether.^4^ It is important to acknowledge that several other factors may also drive RDT use such as provider preference, availability of tests and costs to patients among other socioeconomic drivers that are not addressed within this study. Finally, routine data do not encompass the entire supply of RDTs; individuals within the population may opt for at-home testing options that are accessible and convenient. For example, RDTs such as pregnancy and HIV have self-testing options that can be obtained from standalone pharmacies and are not restricted to health facilities.^58 59^ This study did not estimate unmet needs across all RDTs available in Kenya, partly because of poor reporting rates and the lack of data to estimate demand. For example, urine chemistry tests are used for diagnosing multiple conditions whereas blood sugar requires repeated testing, the frequency of which varies across individuals; such data were not available.

## Conclusion

At the national level, the estimates reveal significant disparities in supply, demand and unmet needs of RDTs, ranging from hundreds of thousands to millions depending on the specific type of RDTs. Subnational estimates further highlight the context-specific variation spanning thousands to millions. These findings emphasise the need for a tailored approach to addressing diagnostic gaps.

Additionally, this study highlights the need for interventions to improve reporting completeness and address geographical variations in supply and testing gaps for specific RDTs in different counties of Kenya. The data availability and reporting patterns across RDTs other than HIV, malaria and VDRL are poor, thereby hampering the overall utility of routine health information systems in disease surveillance and monitoring of diagnostic test availability. In the quest towards UHC, it is necessary to improve facility data records and more accurately estimate demand to quantify unmet needs promptly.

## supplementary material

10.1136/bmjopen-2023-081241 online supplemental file 1

## Data Availability

Data may be obtained from a third party and are not publicly available.
