# Supplementary material for: Evaluating the gap in rapid diagnostic testing: insights from subnational Kenyan routine health data
Source: BMJ Open. 2024 Aug 19;14(8):e081241. doi: 10.1136/bmjopen-2023-081241 (PMC11337709; doi:10.1136/bmjopen-2023-081241)

Supplementary File

Table S1. Summary of the 19 Rapid Diagnostic Tests reported on MoH 706 tool of Kenya's HMIS (DHIS2).

RDTs	WHO EDL classification	Disease outcome	Common	Expected level of reporting
HIV	Sexually transmitted infections	HIV	Y	Level 2-6
Venereal disease research laboratory (VDRL)	Sexually transmitted infections	Syphilis	Y	Level 2-6
Urine Chemistry	Clinical chemistry	UTI, Kidney Disease	Y	Level 2-6
Human chorionic gonadotropin (HCG)	Clinical chemistry	Pregnancy	Y	Level 2-6
Blood Sugar	Clinical chemistry	Diabetes	Y	Level 2-6
Malaria RDT	Bacteriology, mycology, and parasitology	Malaria	Y	Level 2-3
H. pylori	Clinical chemistry	Peptic Ulcers	Y	Level 2-6
Blood Group	Haematology	Blood Transfusion	Y	Level 2-6
HB estimation	Haematology	Anemia	Y	Level 2-6
Rheumatoid factor	Clinical chemistry	Rheumatoid Arthritis	Y	Level 2-6
Brucella	Clinical chemistry	Brucellosis	Y	Level 2-6
Anti streptolysin o titer (ASOT)	Clinical chemistry	Strep Infection	Y	Level 4-6
CHEW Malaria RDTs	Bacteriology, mycology, and parasitology	Malaria	Y	Level 1
Hepatitis B test*	Clinical chemistry	Hepatitis B	Y	Level 2-6
Treponema Pallidum Hemagglutination (TPHA)	Sexually transmitted infections	Syphilis	N	Level 4-6
Cryptococcal Antigen (CRAG) test**	Clinical chemistry	Cryptococcal Meningitis	Y	Level 2-6
Hepatitis A test *	Clinical chemistry	Hepatitis A	Y	Level 2-6
Hepatitis C test*	Clinical chemistry	Hepatitis C	Y	Level 2-6
Oral Glucose Tolerance Test (OGTT)	Clinical chemistry	Diabetes	N	Level 2-6

**Hep A & C need a medical officer to interpret results hence not commonly found in Levels 2 & 3. Hep B is a priority test for ANC and should be performed more often in primary facilities. **The CRAG test is mostly available at facilities that offer antiretroviral therapy.*

Figure S1. Kenya's 47 counties (black), water bodies protected areas and population density.

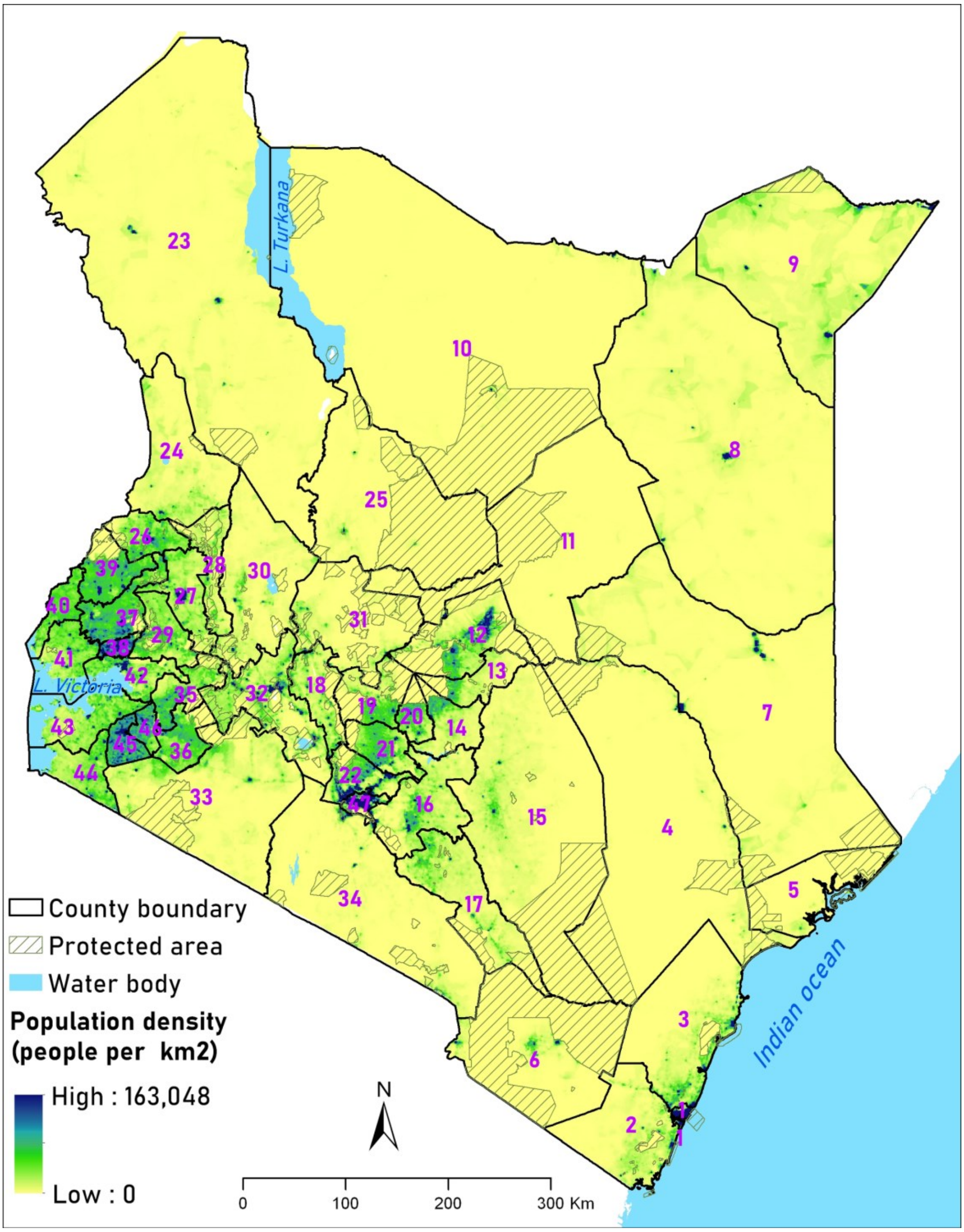

Mombasa [1], Kwale [2], Kilifi [3], Tana River [4], Lamu [5], Taita Taveta [6], Garissa[7], Wajir [8], Mandera [9], Marsabit [10], Isiolo [11], Meru [12], Tharaka-Nithi [13], Embu [14], Kitui [15], Machakos [16], Makueni [17], Nyandarua [18], Nyeri [19], Kirinyaga [20], Murang'a [21], Kiambu [22], Turkana [23], West Pokot [24], Samburu [25], Trans Nzoia [26], Uasin Gishu [27], Elgeyo-Marakwet [28], Nandi [29], Baringo[30],Laikipia [31], Nakuru [32], Narok [33], Kajiado [34],Kericho[35], Bomet [36], Kakamega [37], Vihiga [38], Bungoma[39], Busia [40], Siaya [41], Kisumu [42], Homa Bay [43], Migori [44], Kisii [45], Nyamira [46], Nairobi [47].

Figure S2. A flow chart summarizing the processes used in to estimate demand, supply, and unmet needs of RDTs using routine data in Kenya.

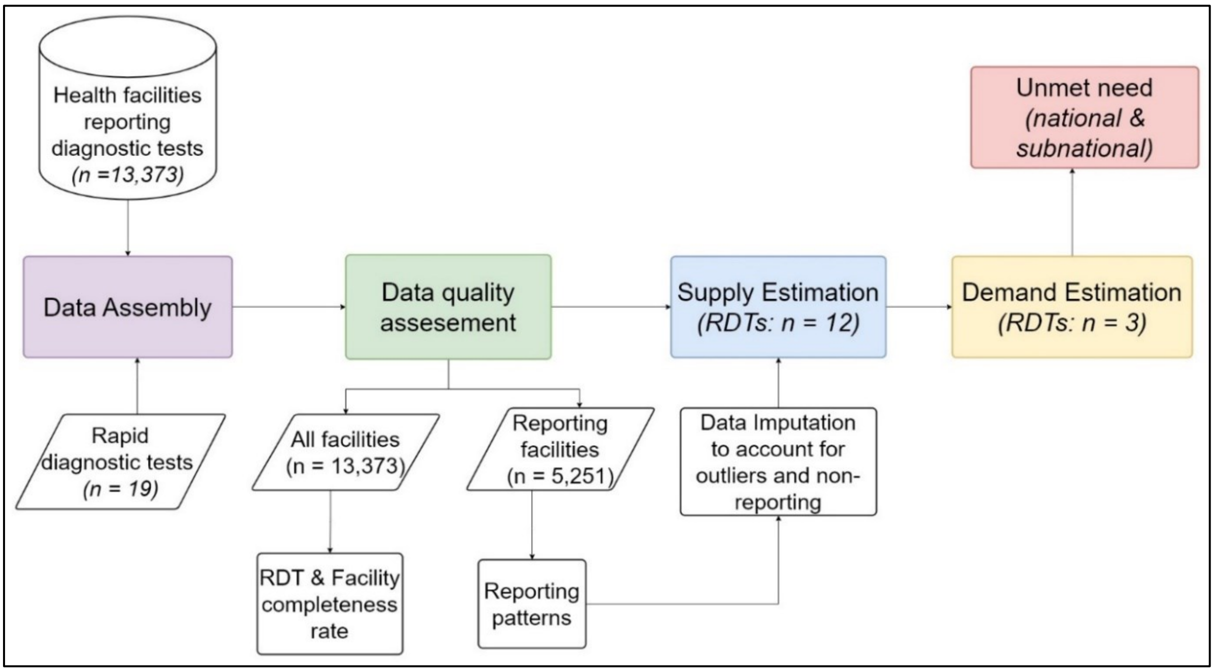

Figure S3. A line graph of Urine Chemistry RDT reporting for six facilities across 36 months (Jan 2018-Dec 2020) indicate outliers identified using the MAD approach (square symbols) .

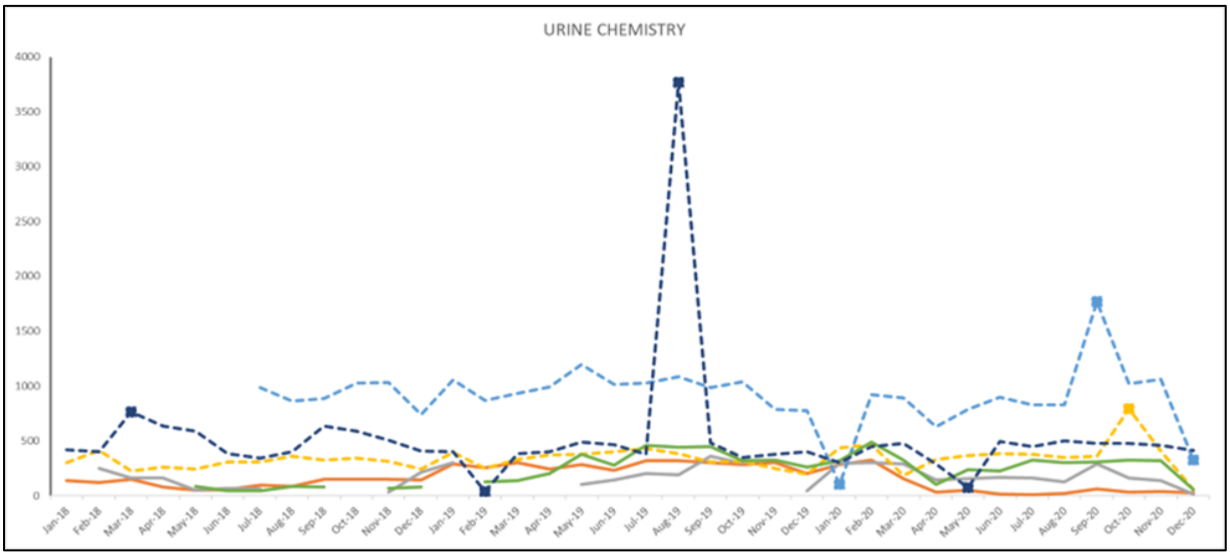

Table S2: A list of under-5 fever-care seeking rates obtained from the Malaria Indicator Survey 2020 (MIS) and HIV testing rate of persons aged 15-64 yrs from the Kenya Aids Indicator Survey (KAIS) used in computing Malaria RDT and HIV RDT demand.

County	Fever-care seeking rate		HIV Testing Rate	
	Malaria Risk Zone	%	Province	%
Baringo	Highland Epidemic Zone\Seasonal	74.0	Rift Valley North	65.7
Bomet	Highland Epidemic Zone	74.0	Rift Valley South	65.7
Bungoma	Lake Endemic\Highland Epidemic Zone	74.0	Western	67.6
Busia	Lake Endemic	56.7	Western	67.6
Elgeyo-Marakwet	Highland Epidemic Zone\Seasonal	74.0	Rift Valley North	65.7
Embu	Seasonal\Low risk	68.8	Eastern South	68.4
Garissa	Seasonal	68.8	North Eastern	71.3
Homa Bay	Lake Endemic	56.7	Nyanza	79.9
Isiolo	Seasonal	68.8	Eastern North	63.1
Kajiado	Seasonal	68.8	Rift Valley South	65.7
Kakamega	Lake Endemic\Highland Epidemic Zone	74.0	Western	67.6
Kericho	Highland Epidemic Zone	74.0	Rift Valley South	65.7
Kiambu	Low risk	63.6	Central	69
Kilifi	Coastal Endemic	64.0	Coast	72.3
Kirinyaga	Low risk	63.6	Central	69
Kisii	Highland Epidemic Zone	74.0	Nyanza	79.9
Kisumu	Lake Endemic	56.7	Nyanza	79.9
Kitui	Seasonal	68.8	Eastern South	68.4
Kwale	Coastal Endemic	64.0	Coast	72.3
Laikipia	Low risk	63.6	Rift Valley North	65.7
Lamu	Coastal Endemic	64.0	Coast	72.3
Machakos	Low risk	63.6	Eastern South	68.4
Makueni	Low risk	63.6	Eastern South	68.4
Mandera	Seasonal	68.8	North Eastern	71.3
Marsabit	Seasonal	68.8	Eastern North	63.1
Meru	Seasonal\Low risk	68.8	Eastern North	63.1
Migori	Lake Endemic	56.7	Nyanza	79.9
Mombasa	Coastal Endemic	64.0	Coast	72.3
Murang'a	Low risk	63.6	Central	69
Nairobi	Low risk	63.6	Nairobi	84.1
Nakuru	Low risk	63.6	Rift Valley South	65.7
Nandi	Highland Epidemic Zone	74.0	Rift Valley South	65.7
Narok	Highland Epidemic Zone	74.0	Rift Valley South	65.7
Nyamira	Highland Epidemic Zone	74.0	Nyanza	79.9
Nyandarua	Low risk	63.6	Central	69
Nyeri	Low risk	63.6	Central	69
Samburu	Seasonal	68.8	Rift Valley North	65.7
Siaya	Lake Endemic	56.7	Nyanza	79.9
Taita Taveta	Coastal Endemic	64.0	Coast	72.3
Tana River	Seasonal	68.8	Coast	72.3
Tharaka-Nithi	Seasonal\Low risk	68.8	Eastern North	63.1
Trans Nzoia	Highland Epidemic Zone	74.0	Rift Valley North	65.7
Turkana	Seasonal	68.8	Rift Valley North	65.7
Uasin Gishu	Highland Epidemic Zone	74.0	Rift Valley South	65.7
Vihiga	Lake Endemic	56.7	Western	67.6
Wajir	Seasonal	68.8	North Eastern	71.3
West Pokot	Highland Epidemic Zone\Seasonal	74.0	Rift Valley North	65.7

Table S3. Summary of the 7 Rapid Diagnostic Tests that were reported by less than 50% of the facilities and excluded from estimating supply, demand, and unmet needs.

Rapid Diagnostic Tests (RDTs)	Reporting facilities, No. (%)*			
	2018-2020 (n=5251)	2018 (n=4381)	2019 (n=4656)	2020 (n=4863)
Anti streptolysin o titer (ASOT)	44.4%	1295 (29.6%)	1587 (34.1%)	1722 (35.4%)
Hepatitis B test	43.0%	1271 (29.0%)	1584 (34.0%)	1782 (36.6%)
Treponema Pallidum Hemagglutination (TPHA)	29.5%	784 (17.9%)	865 (18.6%)	888 (18.3%)
Cryptococcal Antigen (CRAG) test	25.0%	739 (16.9%)	732 (15.7%)	789 (16.2%)
Hepatitis A test	21.8%	482 (11.0%)	593 (12.7%)	641 (13.2%)
Hepatitis C test	21.5%	594 (13.6%)	756 (16.2%)	758 (15.6%)
Oral Glucose Tolerance Test (OGTT)	9.6%	240 (5.5%)	230 (4.9%)	271 (5.6%)

**The proportion of reporting facilities per RDT was computed per year and across 36 months as the number of facilities reporting RDT out of those reporting test volumes > 0 (reporting facilities).*

Figure S4. Geographical variation in the proportion of RDT reports received from those expected to be submitted across the study period (2018-2020) by level. The 16 RDTs included are: VDRL (Venereal disease research), HCG (Human chorionic gonadotropin), Blood sugar, Malaria RDT, H. pylori, Blood grouping, HB Estimation, Rheumatoid Factor, Brucella, ASOT (Antistreptolysin O (ASO) titer test), CHEW Malaria RDTs, TPHA (Treponema Pallidum Hemagglutination), CRAG (Cryptococcal Antigen), Hepatitis A, Hepatitis C, and OGTT (Oral Glucose Tolerance Test).

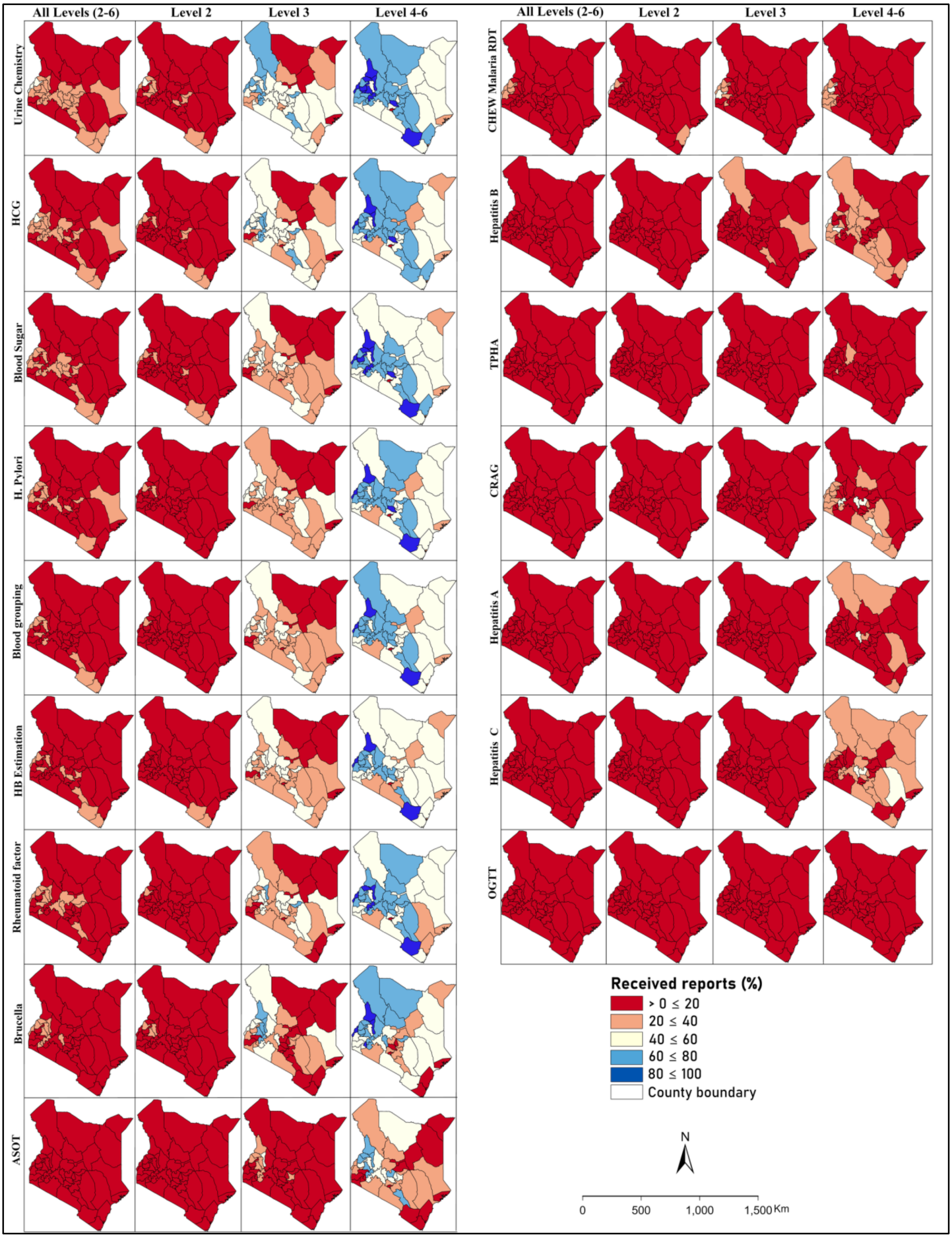

Figure S5A. Geographic variation in supply for Urine Chemistry, HCG, Blood sugar and H. Pylori per year among all reporting facilities.

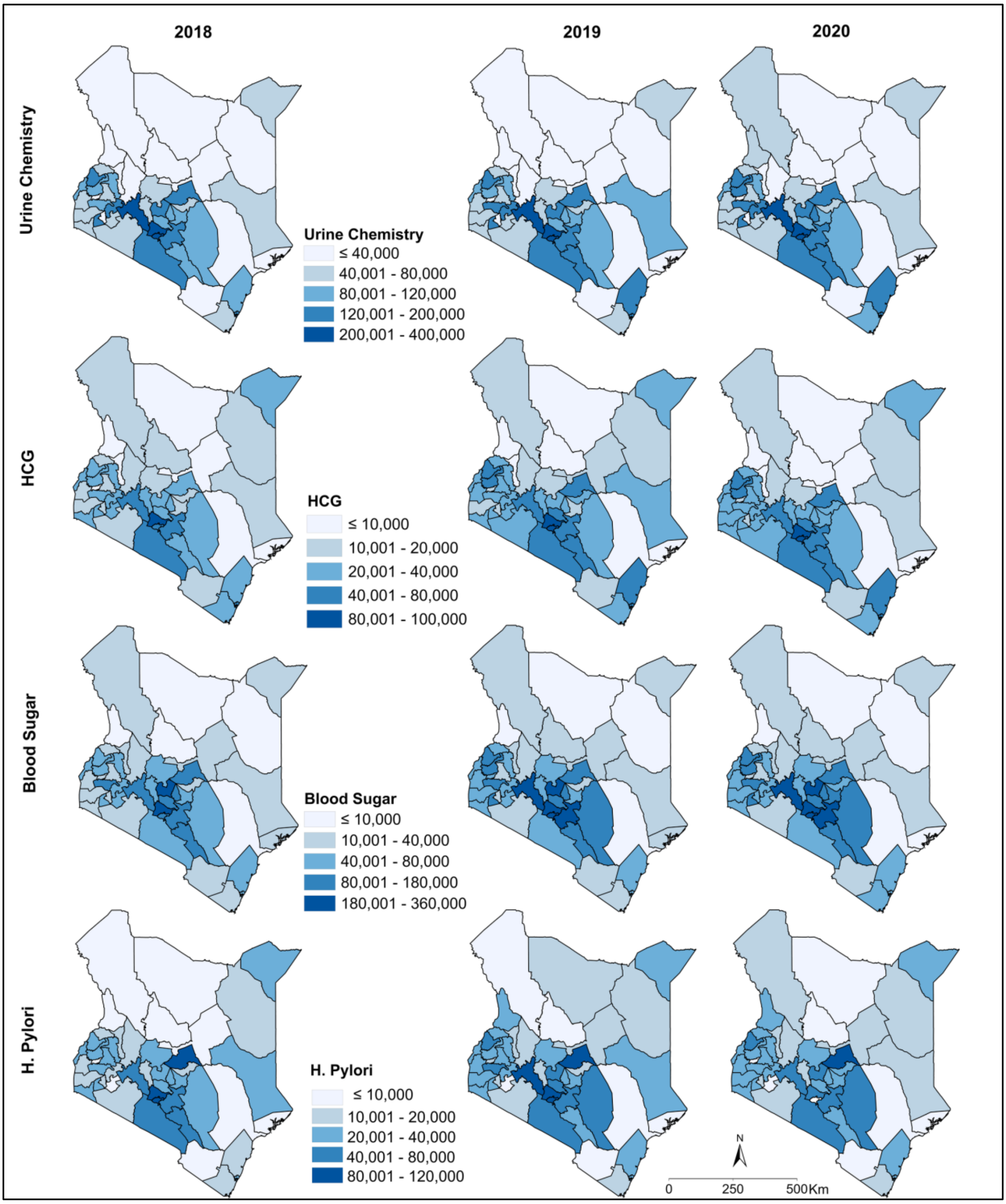

Figure S5B. Geographic variation in supply for Blood Grouping, HB Estimation, Rheumatoid Factor and Brucella per year among all reporting facilities.

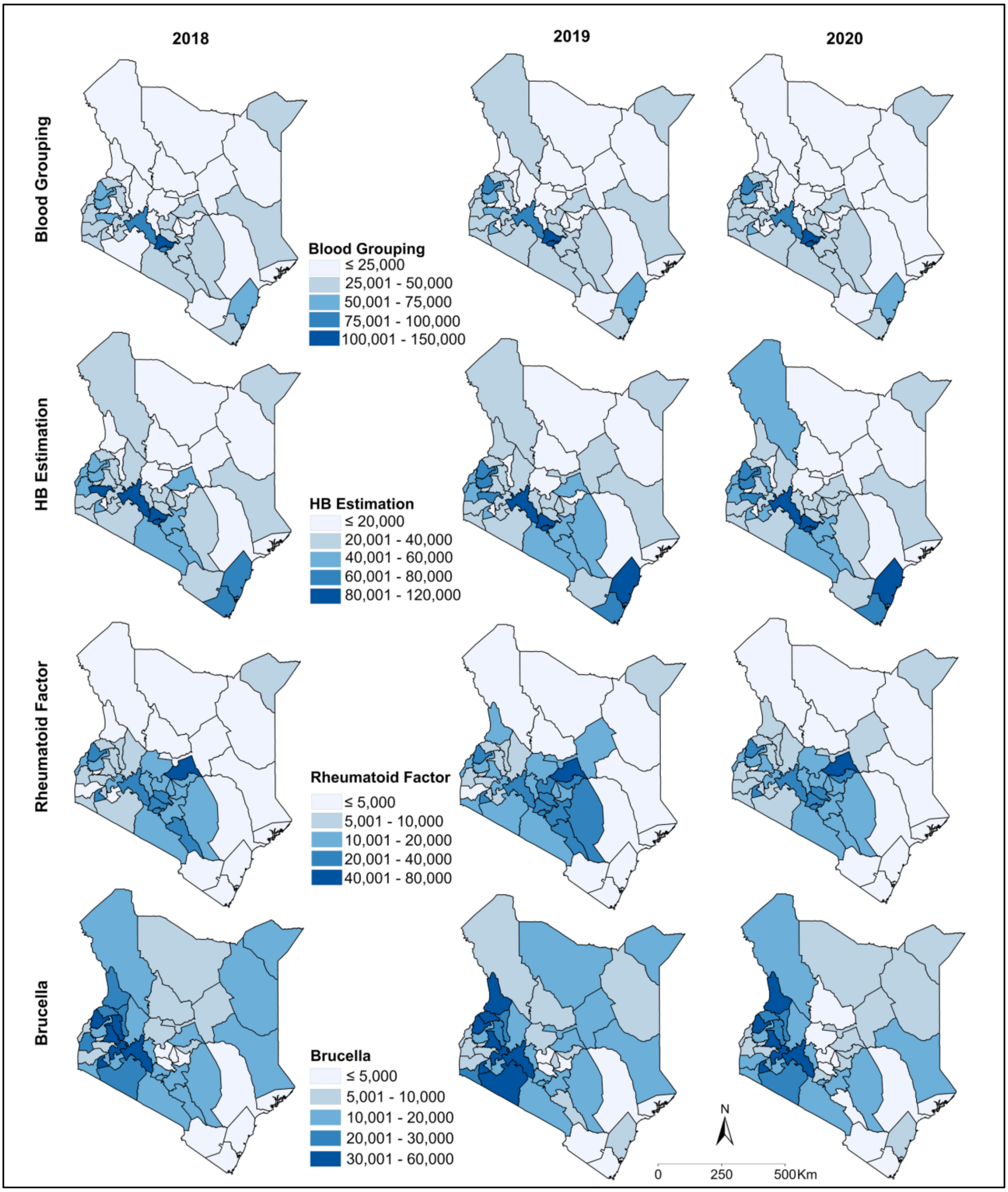

Supplement: online supplemental file 1 [file bmjopen-14-8-s001.pdf]
