# Supplementary material for: Evaluating the gap in rapid diagnostic testing: insights from subnational Kenyan routine health data
Source: BMJ Open. 2024 Aug 19;14(8):e081241. doi: 10.1136/bmjopen-2023-081241 (PMC11337709; doi:10.1136/bmjopen-2023-081241)
Supplement: Supplementary file 2 [file bmjopen-2023-081241.reviewer_comments.pdf]

PEER REVIEW HISTORY

BMJ Open publishes all reviews undertaken for accepted manuscripts. Reviewers are asked to complete a checklist review form and are provided with free text boxes to elaborate on their assessment. These free text comments are reproduced below.

ARTICLE DETAILS

**TITLE (PROVISIONAL)**	Evaluating the Gap in Rapid Diagnostic Testing: Insights from Subnational Kenyan Routine Health Data
**AUTHORS**	Robert, Bibian; Moturi, Angela; Bahati, Felix; Macharia, Peter; Okiro, Emelda

VERSION 1 - REVIEW

**REVIEWER NAME**	Mukonka, Victor M
**REVIEWER AFFILIATION**	High Commission of the Republic of Zambia, Counsellor Health
**REVIEWER CONFLICT OF INTEREST**	None
**DATE REVIEW RETURNED**	26-Jan-2024

**GENERAL COMMENTS**	Generally it is a good paper, however the title is misleading, it mentions geospatial, but there is no single mention of geospatial analysis in the manuscript and no spatial analysis was done apart from availability of RDTs, The abstract need to be very clear on methods and include setting and data analysis. In the introduction, need to come out very clearly on gap analysis, research question and the aim of the study. Methods need major revision to include study setting, period during which data understudy was collected, inclusion criteria, description on data analysis, which statistical package/soft ware used, how the analysis was done. Let the results be presented in the standard format for reporting descriptive studies. In the current form the discussion is quite weak until the results section is redone. There is also need to discuss inherit limitations of this study as the administration of RDTs depends on a myriad of factors including physician preference, patient consent, regional demands etc. This confounders have not been adequately addressed. In addition, the baseline distribution of the RDTs need to be clearly stated, including historical trend to enable comparison of various regions I strongly recommend they address these concerns and resubmit..

**REVIEWER NAME**	Wiens, Kirsten E.
**REVIEWER AFFILIATION**	Temple University
**REVIEWER CONFLICT OF INTEREST**	No competing interests
**DATE REVIEW RETURNED**	29-Jan-2024

**GENERAL COMMENTS**	In this manuscript, Robert et al use multiple types of health data to estimate the unmet need for rapid diagnostic tests in Kenya. This is an important topic, and the authors use a creative and thoughtful approach to synthesize data to address the question. There are some components of the methods need to be clarified and/or further justified before publication, which I have detailed below.

	MAIN COMMENTS<br><br>1) The authors need to provide further justification in how they handle non-reports or missing reports and consider including sensitivity analyses that address this potential source of bias. Specifically:<br>• Lines 161-162, facilities that are missing reports are considered non-reporting instead of true zeros. I understand that this assumption needs to be made, but could the authors provide more context into how this limitation may bias their results? How many of these areas do they expect to be truly zeros for certain indicators?<br>• On lines 232-236, facilities with at least 1 report that are missing reports in any other months are not considered non-reporting in those months (as above) or as true zeros, but rather as missing data and filled in. Could there not be true zeros in a month, for example, if tests ran out? Does changing this assumption alter the conclusions or policy decisions that may be drawn from this study?<br><br>2) I'm a bit concerned about how outliers are handled in calculating supply. Median absolute deviation is used to identify outliers. Are they then all excluded? Please clarify. In addition, please provide justification for excluding outliers. Since this is a sub-national analysis, I expect wide variation in supply and reporting rates and very high or low values may represent true variation in the data. Unless there are strong reasons to believe that these are data entry errors, I suggest including these in the full analysis. If there is good justification for removing outliers, then a plot or table should be added in the supplement to help understand how this dropped data may influence results.<br><br>3) Clarification is needed in how demand was calculated for the malaria and HIV RDTs:<br>• Please provide the formula including definitions of each component, as is done for the VDLR tests.<br>• Please clarify if a single rate was used across the country for care seeking, or if regional or county-level numbers were used. These rates should also be explicitly reported and cited in the text or included in a supplementary table.<br>• Please clarify how total/estimated number of people in each county was considered in calculating county-level demands.<br><br>ADDITIONAL COMMENTS<br><br>• Lines 200-202. Please include how were the "expected" reports calculated.<br>• Line 256. Suggest replacing "hypothesized" with "assumed".<br>• What software was used to manage the data and create the maps? Suggest including this in the methods.<br>• Lines 416-418. Can the authors be more specific in how these results could inform policy? If there are no consistent differences between regions, how should future RDT distribution be prioritized? What additional information is needed?<br>• The authors should also expand in the discussion on how changes since 2020 have likely altered their findings, and how this should be accounted for in policy decisions.

VERSION 1 – AUTHOR RESPONSE

Response to Reviewers	
**Reviewer 1**	**Response**
i) Title is misleading - it mentions geospatial, but there is no single mention of geospatial analysis in the manuscript and no spatial analysis was done apart from availability of RDTs.	We thank the reviewer for this comment. For clarity, the title has been edited to "Evaluating the Gap in Rapid Diagnostic Testing: Insights from Subnational Kenyan Routine Health Data." lines (1-2).
ii) The abstract need to be very clear on methods and include setting and data analysis	The study setting is included see line (30 -31).<br><br>The methods part of the abstract has been rewritten to clearly summarise the data quality assessment done, how supply, demand, and unmet needs were estimated lines (32-40)
iii) In the introduction, need to come out very clearly on gap analysis, research question and the aim of the study	The introduction has been edited for clarity in lines 109-113.
iv) Methods need major revision to include study setting, period during which data understudy was collected, inclusion criteria, description on data analysis, which statistical package/software used, how the analysis was done.	**Setting**<br><br>Under methods: settings, we have introduced two subheadings. One on country setting and healthcare system (130-138) and the other on the Diagnostic landscape, which gives a comprehensive overview of the study setting in the context of diagnostics relevant to the study, provided in lines (140-149).<br><br>**Study period.**<br><br>The study period had been stated in lines 180. We have now included this earlier in the manuscript lines (149).<br><br>**Inclusion criteria**<br><br>The different inclusion criteria used in this study have been stated under their respective subheadings including:<br><br>i. Facilities expected to report diagnostic tests -Here we included all health facilities excluding medical stores, depots, treatment, and rehabilitation centres (170-175).<br><br>ii. Subset of reporting facilities - facilities that submitted at least one test report), totalling 5,231(39.0%), was included was defined (176-180).

	iii. RDTs included in supply estimation – Only 12 RDTs that were reported by at least 50% of health facilities were included in supply estimation lines (246-249.) This was because of the high rate of non-reporting by health facilities; thus, a pragmatic 50% cutoff was applied (231 -234).<br><br>iv. Addressing missingness: Different approaches to account for non-reporting was applied for two sets of facilities: Type 1 that included those reporting at least nine months of diagnostic data per year and type 2 those reporting 1 to 8 months of diagnostic data (type 2) per year lines(252-261). This was to avoid bias since the two types of facilities were systematically different lines (255-256).<br><br>v. RDTs used for demand and unmet needs computation- Only three RDTs (HIV, VDRL, and Total Malaria RDT), were included in the analysis. These were the only RDTs whose data presented minimal ambiguity in demand estimation lines (263-267).<br><br>**Data analysis**<br><br>A subheading indicating the beginning of the analysis has been included in line 215 and description of each step of the analysis detailed from lines 216- 316.<br><br>**Statistical package/software**<br><br>Thank you for highlighting this oversight, R software environment (Version 4.3) and StataCorp. 2021 were the statistical software used in the analysis and results were mapped in ArcGIS Pro 3.0.3 This has now been included in lines(314 - 316).
v) Let the results be presented in the standard format for reporting descriptive studies. In the current form the discussion is quite weak until the results section is redone.	Thank you for the suggestion the results have been revised see lines 333 to 477.
vi) There is also need to discuss inherit limitations of this study as the administration of RDTs depends on a myriad of factors including physician preference,	We agree that there are many competing factors that affect RDT availability and use. In this study, we focused on detailing specific, measurable factors supported by the

	patient consent, regional demands etc. These confounders have not been adequately addressed.	literature and evident in our findings. These include disease prevalence as a driver of demand (lines 515-517), programmatic priorities (lines 517-519), legal environments affecting test availability (lines 541-545), and availability of self-testing options line (560-562).<br><br>We acknowledge that other factors are not considered and are listed in lines 620-622 including those mentioned by the reviewer
vii)	The baseline distribution of the RDTs need to be clearly stated, including historical trend to enable comparison of various regions I strongly recommend they address these concerns and resubmit.	Our study covers a period of 3 years from 2018 -2020 providing insights into historical trends. We have also contextualised areas that are expected to have higher or lower tests based on disease burden e.g. lines (515-519). There is limited historical data before 2018 that could be used for this analysis hence this study provide baseline data for future studies.
**Reviewer 2**<br><br>In this manuscript, Robert et al use multiple types of health data to estimate the unmet need for rapid diagnostic tests in Kenya. This is an important topic, and the authors use a creative and thoughtful approach to synthesize data to address the question. There are some components of the methods need to be clarified and/or further justified before publication, which I have detailed below.		
**1.**	**The authors need to provide further justification in how they handle non-reports or missing reports and consider including sensitivity analyses that address this potential source of bias. Specifically:**	We thank the reviewer for this comment and we have addressed the specifics in 1(a) and 1(b) below.
	a) Lines 161-162, facilities that are missing reports are considered non-reporting instead of true zeros. I understand that this assumption needs to be made, but could the authors provide more context into how this limitation may bias their results? How many of these areas do they expect to be truly zeros for certain indicators?	Yes, facilities with missing reports (blanks) across the entire study period were considered non-reporting, as we could not distinguish whether they represented true zeros or non-reporting or were due to a lack of diagnostic capacity. This challenge primarily arises from the design of the DHIS2 system whereby zero values entered in the system are converted to blank reports. This statement has now been included in lines (182-184) for clarity.<br><br>We agree that true zeros may exist but as a conservative measure we consider facilities that do not make a single report of testing across the entire study period as 'non-reporting'. As this issue also applies to reporting facilities, there could be potential bias introduced in the values that could lead to underestimating or overestimating supply estimates. We have detailed this potential limitations in our study lines (614-620)

	It was not possible to give an exact measure of where to expect true zeros from the system itself due to the immediate transformation of zeros to blanks in DHIS2. Such an assessment would require external validation with the original records from hospitals which is beyond the scope of this study.<br><br>Several studies using Kenya's DHIS2 data have encountered this challenge. The studies include:<br><br>1. Bahati, F., Mcknight, J., Swaleh, F., Malaba, R., Karimi, L., Ramadhan, M., Kiptim, P. K., Okiro, E. A., & English, M. (2022). Reporting of diagnostic and laboratory tests by general hospitals as an indication of access to diagnostic laboratory services in Kenya. PLOS ONE, 17(4), e0266667. https://doi.org/10.1371/journal.pone.0266667<br><br>2. Wambua, S., Malla, L., Mbevi, G., Kandiah, J., Nwosu, A.-P., Tuti, T., Paton, C., Wambu, B., English, M., & Okiro, E. A. (2022). Quantifying the indirect impact of COVID-19 pandemic on utilisation of outpatient and immunisation services in Kenya: A longitudinal study using interrupted time series analysis. BMJ Open, 12(3), e055815. https://doi.org/10.1136/bmjopen-2021-055815<br><br>3. Maina, J. K., Macharia, P. M., Ouma, P. O., Snow, R. W., & Okiro, E. A. (2017). Coverage of routine reporting on malaria parasitological testing in Kenya, 2015–2016. Global Health Action, 10(1), 1413266. https://doi.org/10.1080/16549716.2017.1413266<br><br>4. Odhiambo, J.N., Sartorius, B. Mapping of anaemia prevalence among pregnant women in Kenya (2016–2019). BMC Pregnancy Childbirth 20, 711 (2020). https://doi.org/10.1186/s12884-020-03380-2<br><br>5. Gesicho, M. B., Were, M. C., & Babic, A. (2020). Data cleaning process for HIV-indicator data extracted from DHIS2 national reporting system: A case study of Kenya. BMC Medical Informatics and Decision Making, 20(1), 293. https://doi.org/10.1186/s12911-020-01315-7

b) On lines 232-236, facilities with at least 1 report that are missing reports in any other months are not considered non-reporting in those months (as above) or as true zeros, but rather as missing data and filled in. Could there not be true zeros in a month, for example, if tests ran out? Does changing this assumption alter the conclusions or policy decisions that may be drawn from this study?	We recognize that true zeros may exist and acknowledge the potential impact of stock-outs and DHIS2 limitations (converting true zeros into blanks) in estimating supply in lines (614-620)<br><br>In our previous work (https://doi.org/10.1186/s12913-023-09296-9) we sought to demystify the reasons behind non-reporting across facilities by triangulating DHIS2 data with Kenya Health facility Assessment survey data. Our finding showed that facilities could have diagnostic capacity and still fail to report in the DHIS2 system and in some cases, there could be true zeros due to stockouts, but this will be captured as blanks by the DHIS2. Therefore, to account for such biases in certain months caused by stockouts or improved reporting a 3-year study period was conducted to provide reliable estimates.<br><br>We believe that the conclusions of the study remain a valid starting point as we highlight key unmet needs that would only be exacerbated by issues such as stock-outs.
2. **I'm a bit concerned about how outliers are handled in calculating supply. Median absolute deviation is used to identify outliers. Are they then all excluded? Please clarify.**	We thank the reviewer for highlighting this gap. Outliers were identified using the MAD approach, converted to missing. All missing values were then filled in prior to calculation of supply lines (238-242 and 252-262). The manuscript has been edited for clarity in lines 240-242
a) In addition, please provide justification for excluding outliers. Since this is a sub-national analysis, I expect wide variation in supply and reporting rates and very high or low values may represent true variation in the data. Unless there are strong reasons to believe that these are data entry errors, I suggest including these in the full analysis. If there is good justification for removing outliers, then a plot or table should be added in the supplement to help understand how this dropped data may influence results.	We agree with the reviewer on the expected variation in supply and reporting rates across different regions and maps on supply shown in figure 4 (line 455) captures this variations across counties. To ensure the robustness of our analysis and avoid masking at subnational level, we used the Median Absolute Deviation (MAD) approach to detect outliers at the most granular level possible which is at the facility level and over 36 months which provided a frame of reference to confirm true outliers based on a facility's previous reporting. Additionally, MAD is more sensitive to outliers compared to methods that use standard deviation and mean absolute deviation because it uses the median, which is a robust measure of central tendency not easily influenced by extreme values.<br><br>MAD is particularly useful in datasets with heavy tails or outliers because it provides a more reliable measure of variability.<br><br>Outliers were converted into missing values and filled in alongside other missing values as described in lines (238-

	242). We have now included a graphic to demonstrate an example of outliers identified in the Supplementary file Figure S3
**3) Clarification is needed in how demand was calculated for the malaria and HIV RDTs:**	All clarifications are addressed in 3a, b and c below.
a) Please provide the formula including definitions of each component, as is done for the VDLR tests.	Thank you for the suggestion. The formula used to compute Malaria and HIV RDT has been included in lines (282 - 285)
b) Please clarify if a single rate was used across the country for care seeking, or if regional or county-level numbers were used. These rates should also be explicitly reported and cited in the text or included in a supplementary table.	Health-seeking rates were available at the regional level, and a single value was applied across counties within each respective regions. This has now been clarified in lines (275-277) for Malaria and lines (290 - 292) for HIV. The rates used are included in the supplementary file Table S2
c) Please clarify how total/estimated number of people in each county was considered in calculating county-level demands.	We used an indirect approach of estimating HIV and Malaria RDT demand using health care-seeking rates. This approach assumes that the proportion that sought care represented the number of tests conducted at the facility and those who did not go to a health facility but would have required a test, represented an unmet indirect demand in the community. Therefore, population was not used. The formula in line (282 - 285) shows how demand was computed.<br><br>This approach was deemed reasonable for obtaining county level demand estimates because:<br><br>1. Lack of data on the actual number of people likely to need a HIV or Malaria RDT test.<br>2. Alternative approach that includes population such multiplying disease prevalence by population only accounts for positive tests results significantly underestimating the actual demand.
**4) Additional Comments**	
i) Lines 200-202. Please include how were the “expected” reports calculated.	The expected reports were computed as the total number of facilities expected to report multiplied by the study period (36 months).This now been included in lines (223-224)
ii) Line 256. Suggest replacing “hypothesized” with “assumed”.	This has now been edited as recommended in line 288.
iii) What software was used to manage the data and create the maps? Suggest including this in the methods.	Thank you for highlighting this. R software environment (Version 4.3) and StataCorp. 2021 were the statistical software used in the analysis and results were mapped in ArcGIS Pro 3.0.3. This has now been included in the methods in lines 314-316

iv)	Lines 416-418. Can the authors be more specific in how these results could inform policy? If there are no consistent differences between regions, how should future RDT distribution be prioritized? What additional information is needed?	We thank the reviewer for this comment, and we have highlighted that existing heterogeneities are a signal of where interventions may be most needed lines 508 - 511. We have elaborated on the need for strengthening routine data as well as demand data to better inform policy in the long term in lines 594-601.

VERSION 2 – REVIEW

**REVIEWER NAME**	Mukonka, Victor M
**REVIEWER AFFILIATION**	High Commission of the Republic of Zambia, Counsellor Health
**REVIEWER CONFLICT OF INTEREST**	None
**DATE REVIEW RETURNED**	30-May-2024

**GENERAL COMMENTS**	Generally the author has addressed the issues raised in the review. Just a few things the author needs to pay attention and work out before final publication, make the aim and objectives very clear and smart.<br>The study aimed to assess and estimate the current supply, demand and unmet needs of RDTs in Kenya using routine health data, but reading through the findings and conclusions the author has drawn, it does not clearly answer the aim and objectives of the study, it still needs attention, can the author answer the following questions at the end of the study; what is the estimate, current supply and the gap, unmet needs of RDTs in Kenya, talk to this and make recommendations. I strongly suggest before publication this is revisited so that there is correlation of study aim, findings and the drawn conclusions of the study.

**REVIEWER NAME**	Wiens, Kirsten E.
**REVIEWER AFFILIATION**	Temple University
**REVIEWER CONFLICT OF INTEREST**	I have no competing interests
**DATE REVIEW RETURNED**	16-Apr-2024

**GENERAL COMMENTS**	The authors have provided a thorough revision and edits. I have no additional major comments.<br><br>Minor editorial comments:<br>- The authors may consider revising the "strengths" points to focus on specific aspects of their methods and data that address knowledge gaps and support their conclusions. As written, it is a bit general.<br>- Equation 1 description should be clarified: Where is j in the equation? Is Yi percentage or proportion or rate? What is the definition or unit of "supply" or Xi?<br>- Equation 2 should also be clarified: Is A a percentage or proportion or rate? Can (1-R) be included in the equation itself instead of S?

	- The authors may consider using the term "completeness" instead of "completeness rate" throughout the text.

VERSION 2 – AUTHOR RESPONSE

<table>
<tr><th colspan="2">Response to Reviewers</th></tr>
<tr><th>Reviewer 1</th><th>Response</th></tr>
<tr><td colspan="2">Generally the author has addressed the issues raised in the review. Just a few things the author needs to pay attention and work out before final publication, make the aim and objectives very clear and smart.</td></tr>
<tr><td>1. The study aimed to assess and estimate the current supply, demand and unmet needs of RDTs in Kenya using routine health data, but reading through the findings and conclusions the author has drawn, it does not clearly answer the aim and objectives of the study, it still needs attention, can the author answer the following questions at the end of the study; what is the estimate, current supply and the gap, unmet needs of RDTs in Kenya, talk to this and make recommendations. I strongly suggest before publication this is revisited so that there is correlation of study aim, findings, and the drawn conclusions of the study.</td><td>We agree that the objectives of the study and conclusion need to align. As such, the estimated current supply, demand and unmet have been presented under the results (see lines 430-505) and well summarized using charts for the estimates at the national level (see Figure 3, lines 774 - 778) and maps to show the variation of these estimates at subnational level in Kenya (see Figure 4, lines 780 - 781) and Figures S5A and S5B in the supplementary file.

We want to refer the reviewer to the following specific sections under results where the estimates the reviewer expects have been discussed in detail. Specifically, on

1) RDT supply estimate (lines 430-443) - Here we highlight key trends in the estimated supply for the 12 RDTs over the three years . The specific annual RDT supply estimates at the national level are shown in Figure 3A (see lines 774 - 778). The subnational supply estimates for HIV, Malaria and VDRL are shown in Figure 4 (lines 780 - 781), while the supply estimates for the remaining 9 RDTS are included in Figures S5A and S5B in the supplementary file.

2) Demand and unmet needs Estimates (lines 447 -462) - We have only discussed national estimates for only 3 RDTs (Malaria, HIV and VDRL) and shown in Figure 3B (see lines 774 - 778). We were not able to generate estimates for the other 9 RDTS due to data limitations as elaborated in lines (279 - 283).

3) Supply, demand, and unmet needs at subnational level (lines 464-505) – As explained in 2 above only subnational estimates for 3 RDTs (Malaria, HIV and VDRL) have been discussed and summarized in Figure 4 (lines 780 – 781).

Given the detailed summary in the results we have included an overview of the estimates in the conclusion as recommended to tie our conclusions to the objectives (see lines 659-663)</td></tr>
<tr><td colspan="2">Reviewer 2
The authors have provided a thorough revision and edits. I have no additional major comments.
Minor editorial comments:</td></tr>
</table>

3. The authors may consider revising the "strengths" points to focus on specific aspects of their methods and data that address knowledge gaps and support their conclusions. As written, it is a bit general:	Thank you for your suggestion.<br>We have now revised the strengths to emphasize on:<br><br>1) Granularity of our analysis by county and RDT, lines (57-59).<br><br>2) The robustness of our data quality checks and methods to account for missingness associated with DHIS2 data. See lines (60-62).<br><br>3) The application of indirect techniques to estimate demand and identify unmet needs particulary in settings where direct demand data is limited. See lines (63-65).
4. Equation 1 description should be clarified: Where is j in the equation? Is Yi percentage or proportion or rate? What is the definition or unit of "supply" or Xi?	J represent RDTs and has now been included in the equation. Y_{ij}% is the health care seeking rate for each RDT (j) and county (i). Supply denoted by X_{ij} is total counts in numbers for each RDT (j) and county (i), hence no units. Description of Equation 1 has now been enhanced. See lines (297 - 300)
5. Equation 2 should also be clarified: Is A a percentage or proportion or rate? Can (1-R) be included in the equation itself instead of S?	The percentage symbol has been added where A% refers to the ANC attendance rate calculated (ANC by skilled provider/recent live births in the last 2 years preceding MIS 2020 survey *100)- this had been updated in the description, lines (318 - 323).<br>Thanks for the suggestion, S has been replaced by (1-R) in the equation and the description has been revised as shown in lines 320 - 323
6. The authors may consider using the term "completeness" instead of "completeness rate" throughout the text.	Thank you for the suggestion the term “completeness” has been adopted throughout the text.
